# Quality of Gerontological Nursing and Ageism: What Factors Influence on Nurses’ Ageism in South Korea?

**DOI:** 10.3390/ijerph18084091

**Published:** 2021-04-13

**Authors:** Eun Hee Hwang, Kon Hee Kim

**Affiliations:** 1 Department of Nursing, Wonkwang University, Iksan 54538, Korea; ehh@wku.ac.kr; 2 College of Nursing, Ewha Womans University, Seoul 03760, Korea

**Keywords:** ageism, intelligence, emotional, anxiety, aging, nursing staff

## Abstract

With the aging of the population, age-related problems are emerging, which has caused age discrimination. Particularly, most nurses care for a large number of old patients in the clinical setting. Nurses’ attitude toward the clients has a significant effect on the quality of nursing, so it is time to identify their perspective to the aged. The quality of nursing could greatly depend on who provides and how to provide care. If older patients meet nursing staff with an ageist perspective, whether intentionally or unintentionally, they experience negative attitudes toward them during served health care, and trust cannot be formed, resulting in a deterioration in the quality of medical services. This study aimed to identify factors influencing nurses’ ageism attitudes. A total of 162 general hospital nurses completed a questionnaire consisting of the Wong and Law Emotional Intelligence Scale (WLEIS), the Contact with Elderly People (CEP), the Anxiety about Aging Scale (AAS), and the Fraboni Scale of Ageism (FSA). Data were analyzed by descriptive statistics, t-test, one-way ANOVA, Scheffé post hoc, Pearson’s correlation coefficients, and stepwise multiple regression using SPSS/WIN 26.0 program. The average score of FSA was 2.63 ± 0.36 (range 1–4); FSA showed a statistical difference according to cohabitation with an old adult in the past (t = 2.42, *p* = 0.017). Factors influencing FSA were the fear of old people (β = 0.34, *p* < 0.001) and fear of loss (β = 0.28, *p* < 0.001) of the AAS; and these variables explained 21.1% of FSA (F = 22.56, *p* < 0.001). Based on these results, the development and application of nursing education focused on intergenerational contact is needed in order to reduce the anxiety about aging and to acquire a high quality of gerontological nursing with a reduction of ageism.

## 1. Introduction

Ahead of entering a post-aged society, interest in old age is increasing along with the rapid increase in the number of older people. On the individual perspective, some older adults experience many problems and crises related to physical, mental, and socioeconomic aspects [1,2]. In South Korea, older adults over the age of 65 use hospitalization or outpatient medical services for 50 days or more of the year. That cost accounts for around 40% of the total medical expenses. Considering that older adults are almost 10% of the recipients of medical insurance, the rate of using medical services is higher than that of other age groups [3]. Medical services have characteristics that are not standardized, and it cannot be predicted what kind of services the client will receive until they see medical staff. For this reason, even if the service is the same, the qualitative difference can appear, greatly depending on the provider and the method of provision. Medical services are mainly provided face-to-face, and interaction between the medical staff and the clients is inevitable. In particular, clients become relatively anxious and dependent due to the characteristic that the professionalism of medical staff is the core of medical services [4]. When meeting medical staff with an ageist perspective, whether intentionally or unintentionally, older people suffer a negative attitude toward themselves in the process of being provided services, and this further affects the formation of trust. In the end, the quality of medical services would be affected [5]. Several previous studies have reported a tendency that students as well as experts in the health care field implicitly or explicitly show ageism and have prejudice and discrimination against older adults [5,6,7]. Older people, even healthy, exposed to chronic stressors associated with age discrimination and ageism experience chronic disease, for example, cardiovascular disease, high mortality, and adverse health outcomes, for instance, memory alteration and increased anxiety [8,9]. Therefore, it is urgent to prepare an alternative to this.

Ageism is social discrimination that can arise because of age [10]. Society intentionally excludes certain age groups from opportunities or resources based on chronological age, and as a result functions as an ideology that justifies social inequality such as sexism [11]. In other words, the prejudice and negative ideas toward older adults are solidified into a structure that leads to discrimination and oppression toward older people [12]. Ageism includes all elements of attitude, such as emotions, perceptions, and behaviors [13]. Emotion is the way we feel toward older adults, perception is whether we are thinking them with prejudice, and behavior refers to discrimination in interpersonal relationships with older adults. Discrimination that arises through the administration of policies or institutions can also be included as an element of ageism [14]. However, prior studies on ageism have been mainly conducted on cognitive factors, and studies on emotions, behaviors, and policy factors are rare due to limitations in progress [15,16,17]. Based on the understanding that pre-recognition of phenomena or humans influences our emotions and behaviors, various aspects such as not only perceptions of ageism but also emotions, behaviors, and policy factors should be studied [18].

As one of the emotional variables, emotional intelligence is the ability to observe, evaluate, and differentiate one’s emotions and feelings from others and use them to change thinking and behavior [19]. It consists of five factors: self-awareness, self-regulation, empathy, self-motivation, and interpersonal relations skills. Emotional intelligence makes it possible to use emotions when performing a specific task so that it shows excellent performance for a job [20]. The authors considered emotional intelligence as a significant variable influencing nurses’ ageism, because emotional intelligence is characterized as overcoming negative reactions by properly recognizing, understanding, and controlling the emotions of not only oneself but also others [21]. Therefore, as the older population increases, nurses who frequently take care of older patients will be able to cope with the negative emotions such as aging anxiety and the stress they receive due to adverse health outcomes associated with aging. As a result, the nurse could provide age-integrative nursing care with positive emotions about older adults. The finding that emotional intelligence significantly reduces stress and burnout in emergency room nurses [22] supports our hypothesis. 

Prejudice and discrimination could be reduced by reinforcing direct contact between members of the two groups and by experiencing positive contact. The experience of contact with other groups is not limited to one experience, and through this, it affects other situations as well [23,24,25]. Some studies found that the more interactions that there were with older adults, the more positive the attitudes were toward aging and older people [26]; and that the frequency of positive contact with older adults influences the perception of older adults [27]. Others reported that professional caregivers who are older and experienced tend to be age-discriminative [28,29]. Although controversial, it is helpful to reduce the problem of prejudice or discrimination against the older population by increasing the frequency of nurses’ contact with older adults; in particular, by making that contact positive and meaningful.

Although aging is a natural change that everyone experiences, people usually perceive aging as negative [30,31], and this can lead to aging anxiety, an emotion that is negative about aging. Aging anxiety is a concept that combines worry and fear about aging [32]. Choosing a lifestyle or opportunity is a major factor influencing attitudes toward aging and determining life in old age [33]. Aging anxiety can impair psychological well-being and acts as a modulator that determines attitudes and behavior toward older adults [34,35]. The more positive the perception or attitude toward older people, the less the discrimination or prejudice there is against them, and positive and valuable experience with older adults is important to understand them and to form a positive attitude [36]. Therefore, it is predicted that the aging anxiety of nurses will have a significant effect on the outcome of gerontological nursing. However, studies related to aging anxiety were limited to age groups or college students.

As the older population increases, the nurses, as professional health care providers, encounter older adults who are healthy or unhealthy more often; and their attitude toward older adults affects nursing outcomes. Ageism, a negative age stereotype, could be affected by various factors including cognitive, emotional, social, and politic aspects; however, the variables identified as factors influencing ageism are very limited [5,6,7,8,9,10,11,12,13,14,15,16,17,18,23,24,25,26,27].

Therefore, considering a variety of aspects of ageism in the nursing area, this study aimed to identify the effects of the emotional intelligence, elderly contact experience, and aging anxiety on ageism of general hospital nurses caring for old adult clients, which are increasing with the rapid increase of the older population.

## 2. Materials and Methods

### 2.1. Design

This descriptive and correlational study aimed to identify how factors such as emotional intelligence, contact experience with old people, and anxiety about aging can influence ageism.

### 2.2. Participants

One hundred sixty-two nurses from two general hospitals at a city of J province in South Korea participated in this study. Participants were working in general wards and special wards. The inclusion criteria were nurses who worked for more 6 months at general hospitals and participated in direct nursing. Two-tailed test and multiple regression analysis were performed using the G*Power program 3.1.9.2 with the medium effect size (f^2^ = 0.15), power 0.85, significance level 0.05, test variable 16, and the number of samples 157. After the convenient selection of 180 nurses, a questionnaire was distributed. Eight nurses dropped out of the study because they refused to participate, and ten nurses who did not complete the questionnaire were excluded, so the study was conducted for the remaining 162 participants.

### 2.3. Measurements

#### 2.3.1. Ageism

Participants’ ageism was measured using a Korean version [37] of the Fraboni Scale of Ageism (FSA), developed by Fraboni, Saltstone, and Hughes [38]. This scale has three subscales, the emotional avoidance (EA), the discrimination, and the stereotype. The FSA is a four-point Likert scale with 18 items, each of which was assigned with a point value between 1 (strongly disagree) and 4 (strongly agree). Scores ranged from 1 to 4; the higher the score, the more negative the ageism attitude. When validating the Korean scale, the Cronbach’s α reliability was 0.85 [37], and it was 0.79 in this study.

#### 2.3.2. Emotional Intelligence

Emotional intelligence was measured using the Wong and Law Emotional Intelligence Scale (WLEIS), developed by Wong and Law [39] and translated in Korean by Kim and Hwang [40]. It has four subscales, the self-emotional appraisal (SEA), the others’ emotional appraisal (OEA), the regulation of emotion (ROE), and the use of emotion (UOE). The WLEIS is a five-point Likert scale with 16 items, each of which was assigned with a point value between 1 (strongly disagree) and 5 (strongly agree). Scores ranged from 1 to 5; the higher the score, the higher the emotional intelligence. The Cronbach’s α reliability was 0.87 when developing the scale [39], 0.91 in Kim and Hwang [40], and 0.88 in this study. 

#### 2.3.3. Contact with Elderly People

For elderly contact uncertainty, a Korean version [37] of the Contact with Elderly People (CEP), developed by Hutchison, Fox, Laas, Matharu, and Urzi [17], was used as the measure. This scale is a seven-point Likert scale with 6 items; each item was assigned a point value between 1 (strongly disagree) and 7 (strongly agree). Scores ranged from 1 to 7; the higher the score, the more positive the experience in contact with older people. The Cronbach’s α reliability was 0.86 [17] when developing the instrument, 0.86 in Kim et al. [37], and 0.86 in this study.

#### 2.3.4. Anxiety about Aging

For measuring anxiety about aging, a Korean translation version [41] of the Anxiety about Aging Scale (AAS), developed by Lasher and Faulkender [35], was used. The AAS is a five-point Likert scale with 20 items in four subscalee: the fear of old people (FOP), the psychological concerns (PC), the physical appearance (PA), the fear of loss (FL). Each item was assigned a point value between 1 (strongly disagree) and 5 (strongly agree). Scores ranged from 1 to 5. The Cronbach’s α was 0.82 [35] when developing the instrument, 0.86 in Kim [41], and 0.86 in this study. 

### 2.4. Data Collection

After approval by the Institutional Review Board (IRB), data were collected from nurses from two general hospitals in April–May 2019. The authors received approval and cooperation from the nursing department in advance and posted a notice of recruitment of participants on the announcement board of each ward in the hospitals. After explaining the purpose and procedure of the study to the nurses who read the notice, decided participation, and gathered at specific places prepared in each hospital, informed consent was obtained from those who agreed to participate in this study, and then a structured questionnaire was distributed. It took about 20 min to respond, and after submitting the questionnaire, a small gift was provided. 

### 2.5. Data Analysis

This study performed data analysis using the SPSS/WIN 26.0 program (IBM, Armonk, NY, USA). The mean and standard deviation of the general characteristics, ageism, emotional intelligence, contact with elderly people, and anxiety about aging were analyzed. Ageism was differentiated according to the participants’ general characteristics and analyzed using t-test and ANOVA; Scheffé post hoc verified post-analysis. Correlations between ageism, contact with elderly people, and anxiety about aging were computed using Pearson’s correlation coefficients. The influences of emotional intelligence, contact with elderly people, and anxiety about aging on ageism were identified via multiple linear regression analysis.

## 3. Results

### 3.1. General Characteristics

Participants were 35.36 ± 8.84 years old; those <29 years old were the most considerable proportion (37.6%). All of participants were female, 51.9% were married, 42.0% had religion, 75.9% perceived their personality introverted, and 74.7% had minimum bachelor-level education. For the position in the hospitals, 82.7% were staff nurses. Thirty-seven percent of participants had <5 years clinical experience; 93.2% had experience of nursing older adult patients. Over half (54.9%) of the nurses reported they had lived with older adults in the past, and 8.6% answered they are currently living with older adults. Seventy-one percent completed the gerontological nursing course while enrolled in nursing school, and 46.3% received gerontological nursing-related education working as a nurse (Table 1).

### 3.2. Participants’ Emotional Intelligence, Contact with Elderly People, Anxiety about Aging, and Ageism

The WLEIS score was 3.43 ± 0.42 for participants. In terms of subscales of the WLEIS, SEA, OEA, ROE, and UOE scores were 3.81, 3.50, 3.33, and 3.07, respectively. The CEP score was 4.00 ± 1.15; as subscales of the CEP, CF and CQ scores were 4.20 and 3.80, respectively. The AAS score was 2.94 ± 0.42; according to its subscales, FOP, FL, PA, and PC scores were 3.14, 2.88, 2.87, and 2.85, respectively. The FSA score was 2.63 ± 0.36, and in the case of its subscales, EA was 2.61, discrimination was 2.52, and stereotype was 2.74 (Table 2).

### 3.3. Differences in Ageism According to General Characteristics

Participants’ FSA was shown to be significantly different for cohabitation with older adults in the past (t = 2.42, *p* = 0.017). In terms of the FSA subscales, emotional avoidance was shown to be different for cohabitation with older adults in the past (t = 2.76, *p* = 0.006); discrimination was different by clinical experience (F = 2.68, *p* = 0.049), however, post-test result was not significant; the antilocution was different for different ages (F = 4.44, *p* = 0.013) and clinical experience (F = 4.93, *p* = 0.003). The post-test showed that the ≥40 age group had higher antilocution scores than those of the <29 age group and that the group with clinical experience <5 years had higher antilocution scores than those of the group with ≥15 years’ experience; these differences were statistically significant (Table 3).

### 3.4. Correlations among Emotional Intelligence, Contact with Elderly People, Anxiety about Aging, and Ageism

Participants’ FSA was shown to be negatively correlated with ROE of WLEIS (r = −0.28, *p* < 0.001) and CEP (r = −0.28, *p* < 0.001) and its subscales, contact frequency (CF) (r = −0.23, *p* = 0.004) and contact quality (CQ) (r = −0.30, *p* < 0.001). The AAS (r = 0.37, *p* < 0.001) and its subscales, FOP (r = 0.38, *p* < 0.001), PA (r = 0.21, *p* = 0.008), and FL (r = 0.34, *p* < 0.001), were positively correlated with the FSA (Table 4). 

### 3.5. Factors Influencing Ageism

In order to identify the influencing factors of the FSA, a stepwise multiple linear regression was performed. ROE, a subscale of the WLEIS; the CEP and its subscales, CF and CQ; the AAS and its subscales, FOP, PA, and FL were used as predictive variables as they had been confirmed for significant correlation. Out of the general characteristics, age, clinical experience, and cohabitation with older adults were imported as they had shown significant differences from the FSA (Table 5). As a result, the subscales of the AAS, FOP (β = 0.34, *p* < 0.001), and FL (β = 0.28, *p* < 0.001) were revealed as significant factors. These variables could explain the FSA by up to 21.1% (F = 22.56, *p* < 0.001). The detection of multicollinearity, residual, and outliers was performed to test the predictive variable’s regression analysis hypothesis. The tolerance was 0.98, and the variance inflation factor (VIF) was 1.02, indicating that there was no multicollinearity issue. Durbin–Watson value was 1.92.

## 4. Discussion

Along with the increase in the population of older people, ageism, which means discrimination against age, has emerged, and negative results in physical, cognitive, emotional, and social aspects have been reported [34]. The number of older adults is increasing rapidly, and their use of medical services is increasing. Nurses are a significant part of health care providers and play a key role. Therefore, this study focused on the level of general hospital nurses’ ageism and its influencing factors. The discussion of major findings of this study is as follows.

The participants’ FSA score in this study was 2.63, which was moderate, similar to the 2.74 score of general hospital nurses in the Bilim and Kutlu study [42], and higher than the 2.39 score in the research of Rababa et al. [43]. By subscale, the EA score was the highest, and similar to the research results of Lee et al. [44]. The EA means wanting to distance from older adults because of negative emotions [37]. In our study, the FSA, FSA subscales, and the EA scores were significantly different depending on cohabitation with older adults in the past, and the scores were significantly higher in the case of no experience of living with them. Among the subscales of the FSA, significant differences were found according to age and clinical experience; and the scores of nurses in their 40 s were significantly higher than those of nurses in their 20s, and it can be seen that the scores of the corresponding area increase as age increases. This is different from the results that there was no significant difference in ageism according to age [45] and that the younger people had severe elder discrimination [46,47]. We found a significant difference in a subscale of the FSA, the discrimination, according to clinical experience; and scores of less than 5 years were significantly lower than those of more than 15 years. It is similar to the result that the ageist score of nurses with less than 5 years of experience was significantly lower than that of those with 5 years or more [48]. This result describes that experienced nurses consider employment in long-term care facilities like nursing homes rather than in acute institutions because it is difficult to meet the physical demands required for their job [48]. Therefore, it is needed to examine whether the result that a higher FSA score of nurses with more clinical experience is due to nurses’ burnout in acute settings.

In our study, the participants’ FSA negatively correlated with ROE of the WLEIS, the CEP and its subscales, the CF and CQ; the AAS and its subscales, the FOP, PA, and FL, positively correlated with the FSA. It is in line with the result that the degree of ageism is associated with the frequency and quality of contact with older adults; the less frequent the contact with older adults and the more negative the quality of contact, the more severe the degree of ageism [26,36,49]. Ageism includes elements such as emotions, perceptions, and behaviors [13], and it can be seen that ageism of general hospital nurses has a significant correlation with emotional variables such as emotional intelligence and aging anxiety. In ageism, since emotions are the way they feel about older people and are influenced by prior perceptions of them [13,18], at this point, efforts to identify the level of general hospital nurses’ emotion and perception are necessary. Then, we can develop interventions that can change our perception of older adults and lead to a change in the emotional aspect.

These correlation results were also confirmed in the factors that influence the ageism of the participants in this study. As a result of multiple regression analysis, the variables that were found to have a significant effect on ageism were FOP and FL, the subscales of the AAS, an emotional variable; the explanatory power of these variables was 21.1%. It is similar to the study results of Liu et al., who reported aging anxiety as an influencing factor of ageism in nurses, and Cooney, Minahan, and Siedlecki, who reported aging anxiety as an influencing factor of ageism in all age groups over 18 years of age [27,50]. The greater the fear of older adults, the greater the fear of loss, and the less anxiety about the physical change by aging, the higher the ageism score. Actually, in the environment of a hospital, nurses frequently meet older patients who often encounter negative changes associated with aging, such as cognitive impairment, or limited rights of health decision-making. It is thought that the higher the degree of anxiety about these situations, the higher the FSA score. Therefore, cultivating correct perceptions of older adults and aging can be considered as an alternative to preventing these consequences from occurring.

In the AAS, FOP refers to the degree of acceptance of older adults and fear of the formation of a relationship with them, and FL is concerning the loss of physical health, decision-making rights, and social roles due to aging [41]. Considering the characteristics of the AAS, nurses are well aware of the negative effects on health and physical changes by aging due to the professional nature of being a health care provider, so the degree of anxiety about them is high. Lee et al. [44] reported that the more the knowledge about older adults, the lower the degree of ageism, and as a result, they had a significant negative correlation, which supports our findings.

However, considering the research results that direct contact with older people improves the accuracy of pre-recognition of older adults and reduces negative prejudice toward them [51], it is somewhat concerned that the emotional dimension variable was identified as a significant influencing factor on the ageism of nurses who frequently encounter older patients in clinical settings. This is because the degree of ageism of the medical staff has a significant effect on the type of treatment recommended for patients and, in turn, the health outcome [52]. According to our findings, compared with the rate of completing gerontological nursing courses at nursing colleges (around 70%), the completion rate of gerontological nursing-related education in clinical settings was low (around 45%). In addition, the meta-analysis research reported that both nurses and nursing students lack knowledge about gerontological nursing and have misconceptions in caring for older adults [53]. It indicates that education for improving perceptions of older people for nurses is needed. Moreover, the previous meta-analysis research reported that education and intergenerational contact are effective interventions to reduce ageism [54], so it would be more effective to increase the frequency of intergenerational contact with education intervention.

Unlike previous studies, age, clinical experience, and cohabitation experience with older adults in the past were not influencing factors in our findings. Based on the previous study [46], which identified that gender is a significant influence factor on ageism, it is thought that the characteristics of the participants, consisting of all females according to convenience sampling, were reflected. Therefore, we suggest follow-up studies on ageism according to general characteristics such as gender and age. In particular, the contact experience with older adults is useful for understanding older people more accurately, reduces negative prejudice against them, and is reported as an influencing factor affecting ageism in many studies [27,37,55], therefore, further expansion and repetitive studies are needed.

This study has the following limitations. As convenience sampling is limited to one country, attention should be paid to the generalization of research results. In addition, there is a possibility that social desirability may be brought about by a self-reported questionnaire, and since the research participants are all women, it is impossible to identify differences according to gender, and the explanatory power of the regression analysis result was only 21.1%. The authors could explain that the low explanatory power resulted from specification errors and the exclusion of detailed characteristics of contact with older adults including quantity and types. Therefore, we suggest considering other variables such as functionality of the older people to which nurses are exposed. Particularly, in the case of ageism, policy aspects must be considered along with perception and emotional aspects, so it is suggested to confirm the policy efforts to resolve ageism in the health care field and their impact.

## 5. Conclusions

In the aging and aged era, ageism is a serious problem in health care, including nursing. Ageism affects various aspects of patients’ health outcomes. In clinical settings, nurses encounter many patients, and the majority of them are old people. However, nursing care does not focus on ageism fully. In order to acquire the high quality of elderly nursing, we identified the degree of ageism and its influencing factors in nurses. Our findings revealed that general hospital nurses have moderate ageism attitudes and sub-scales of the anxiety of aging were significant influencing factors on ageism. It is necessary to lower the aging anxiety to move closer to a solution for ageism in nursing. Some variables, including age, clinical experience, and cohabitation with the aged, were not influencing factors, unlike in previous studies. Ageism is not a simple concept and consists of perception, emotion, and policy. Therefore, various aspects of nurses’ ageism should be investigated. Based on this study, nursing education intervention focusing on the appropriate perception of aging and older adults could be considered. These efforts will be the basis for the development of gerontological nursing by providing quality nursing care based on age-integrated perception, not on age-specific separation and conflict. 

## Figures and Tables

**Table 1:** Participants’ general characteristics. (*N* = 162).

Characteristics	Categories	*n* (%), Mean ± SD
Age (year)		35.36 ± 8.84
	<29	61 (37.6)
	30–39	51 (31.5)
	≥40	50 (30.9)
Gender	Female	162 (100.1)
Marriage	Yes	84 (51.9)
	No	78 (48.1)
Education	Associate degree	41 (25.3)
	≥Bachelor degree	121 (74.7)
Religion	Yes	68 (42.0)
	No	94 (58.0)
Perceived personality	Introvert	123 (75.9)
	Extrovert	39 (24.1)
Position	Staff nurse	134 (82.7)
	Nursing manager	28 (17.3)
Clinical experience (year)		10.37 ± 8.96
	<5.0	60 (37.0)
	5.0–9.9	35 (21.6)
	10–14.9	22 (13.6)
	≥15.0	45 (27.8)
Nursing experience of older patients	Yes	151 (93.2)
	No	11 (6.8)
Cohabitation with older adults	Yes	14 (8.6)
	No	148 (91.4)
Cohabitation with older adults in the past	Yes	89 (54.9)
	No	73 (45.1)
Completion of the gerontological nursing course at nursing school	Yes	115 (71.0)
	No	47 (29.0)
Completion of gerontological nursing education as a nurse	Yes	75 (46.3)
	No	87 (53.7)

**Table 2:** Emotional intelligence, contact with elderly people, anxiety about aging, and ageism.

(*N* = 162)	Possible Range	Mean ± SD	Min	Max
Emotional intelligence	1–5	3.43 ± 0.42	3	5
Self-emotional appraisal	1–5	3.81 ± 0.57	2	5
Others’ emotional appraisal	1–5	3.50 ± 0.49	2	5
Regulation of emotion	1–5	3.33 ± 0.57	2	5
Use of emotion	1–5	3.07 ± 0.65	1	5
Contact with elderly people	1–7	4.00 ± 1.15	1	7
Contact frequency	1–7	4.20 ± 1.25	1	7
Contact quality	1–7	3.80 ± 1.26	1	7
Anxiety about aging	1–5	2.94 ± 0.42	1	4
Fear of old people	1–5	3.14 ± 0.60	1	5
Psychological concerns	1–5	2.85 ± 0.51	1	4
Physical appearance	1–5	2.87 ± 0.55	1	4
Fear of loss	1–5	2.88 ± 0.66	1	4
Ageism	1–4	2.63 ± 0.36	2	4
Emotional avoidance	1–4	2.61 ± 0.50	1	4
Discrimination	1–4	2.52 ± 0.40	1	4
Stereotype	1–4	2.74 ± 0.45	2	4

**Table 3:** Differences in ageism according to general characteristics. (*N* = 162).

Characteristic		Ageism							
		Total		Avoidance		Discrimination		Antilocution	
		Mean ± SD	t/F(*p*) Scheffé	Mean ± SD	t/F(*p*) Scheffé	Mean ± SD	t/F(*p*) Scheffé	Mean ± SD	t/F(*p*) Scheffé
Age (year)	<29 ^a^	2.62 ± 0.38	0.23(0.794)	2.73 ± 0.53	2.68 (0.072)	2.45 ± 0.36	1.64 (0.197)	2.63 ± 0.45	4.44 (0.013)
	30–39 ^b^	2.62 ± 0.40		2.53 ± 0.53		2.58 ± 0.42		2.75 ± 0.45	a < c
	≥40 ^c^	2.66 ± 0.30		2.55 ± 0.41		2.54 ± 0.42		2.88 ± 0.44	
Marriage	Yes	2.64 ± 0.36	0.37 (0.716)	2.55 ± 0.48	0.67 (0.119)	2.56 ± 0.40	1.53 (0.128)	2.81 ± 0.49	1.78 (0.077)
	No	2.62 ± 0.37		2.67 ± 0.52		2.47 ± 0.39		2.68 ± 0.41	
Education	ADN ^1^	2.64 ± 0.39	0.30 (0.763)	2.64 ± 0.55	0.51 (0.613)	2.56 ± 0.39	0.75 (0.458)	2.72 ± 0.46	0.48 (0.632)
	≥BSN ^2^	2.62 ± 0.35		2.60 ± 0.49		2.50 ± 0.40		2.75 ± 0.45	
Religion	Yes	2.62 ± 0.32	0.36 (0.716)	2.55 ± 0.50	1.25 (0.214)	2.53 ± 0.37	0.28 (0.778)	2.77 ± 0.48	0.53 (0.596)
	No	2.64 ± 0.39		2.65 ± 0.50		2.51 ± 0.42		2.73 ± 0.44	
Perceived personality	Introvert	2.63 ± 0.36	0.04 (0.966)	2.61 ± 0.49	0.03 (0.975)	2.50 ± 0.41	0.96 (0.340)	2.76 ± 0.46	0.76 (0.447)
	Extrovert	2.63 ± 0.38		2.61 ± 0.55		2.57 ± 0.35		2.70 ± 0.44	
Position	Staff	2.62 ± 0.37	0.32 (0.749)	2.62 ± 0.52	0.27 (0.786)	2.53 ± 0.40	0.97 (0.336)	2.72 ± 0.46	1.84 (0.068)
	Manager	2.65 ± 0.31		2.59 ± 0.42		2.45 ± 0.38		2.89 ± 0.42	
Clinical experience (year)	<5.0 ^a^	2.56 ± 0.38	1.15 (0.331)	2.65 ± 0.56	0.37 (0.775)	2.41 ± 0.38	2.68 (0.049)	2.60 ± 0.45	4.93 (0.003)
	5.0–9.9 ^b^	2.64 ± 0.37		2.59 ± 0.50		2.61 ± 0.35		2.73 ± 0.44	a < d
	10–14.9 ^c^	2.69 ± 0.35		2.66 ± 0.41		2.60 ± 0.41		2.81 ± 0.48	
	≥15.0 ^d^	2.68 ± 0.32		2.56 ± 0.47		2.55 ± 0.43		2.92 ± 0.40	
Nursing experience of aged patients	Yes	2.63 ± 0.36	0.26 (0.792)	2.60 ± 0.51	0.89 (0.375)	2.50 ± 0.39	1.68 (0.096)	2.76 ± 0.45	1.75 (0.082)
	No	2.66 ± 0.32		2.74 ± 0.39		2.71 ± 0.42		2.52 ± 0.41	
Cohabitation with the aged	Yes	2.47 ± 0.34	1.76 (0.080)	2.42 ± 0.37	1.50 (0.135)	2.46 ± 0.40	0.58 (0.564)	2.54 ± 0.40	1.82 (0.071)
	No	2.64 ± 0.36		2.63 ± 0.51		2.52 ± 0.40		2.76 ± 0.45	
Cohabitation with the aged in the past	Yes	2.57 ± 0.38	2.42 (0.017)	2.51 ± 0.49	2.76 (0.006)	2.49 ± 0.38	1.08 (0.280)	2.70 ± 0.48	1.38 (0.168)
	No	2.70 ± 0.32		2.73 ± 0.49		2.55 ± 0.42		2.80 ± 0.41	
Completion of the GN ^3^ course at nursing school	Yes	2.64 ± 0.36	0.46 (0.649)	2.63 ± 0.48	0.93 (0.356)	2.54 ± 0.40	1.24 (0.215)	2.72 ± 0.47	1.02 (0.311)
	No	2.61 ± 0.35		2.55 ± 0.55		2.46 ± 0.38		2.80 ± 0.42	
Completion of GN ^3^ education	Yes	2.59 ± 0.36	1.37 (0.172)	2.57 ± 0.49	0.96 (0.339)	2.50 ± 0.43	0.52 (0.607)	2.68 ± 0.46	1.64 (0.103)
	No	2.66 ± 0.36		2.65 ± 0.51		2.53 ± 0.37		2.80 ± 0.44	

^1^ Associate Degree in Nursing. ^2^ Bachelor of Science in Nursing. ^3^ Gerontological Nursing.

**Table 4:** Correlation among emotional intelligence, elderly contact experience, anxiety about aging, and ageism. (*N* = 162).

r (*p*)		EI ^1^					CEP ^6^			AA ^9^					Ageism
		Total	SEA ^2^	OEA ^3^	UOE ^4^	ROE ^5^	Total	CF ^7^	CQ ^8^	Total	FOP ^10^	PC ^11^	PA ^12^	FL ^13^	
EI ^1^	Total	1	0.62 (<0.001)	0.69 (<0.001)	0.82 (<0.001)	0.77 (<0.001)	0.15 (0.065)	0.06 (0.440)	0.20 (0.009)	−0.32 (<0.001)	−0.27 (<0.001)	−0.41 (<0.001)	−0.24 (0.002)	−0.05 (0.493)	−0.09 (0.282)
	SEA ^2^		1	0.28 (<0.001)	0.27 (<0.001)	0.25 (0.001)	0.04 (0.607)	−0.04 (0.615)	0.11 (0.149)	−0.23 (0.004)	−0.16 (0.048)	−0.26 (0.001)	−0.18 (0.019)	−0.08 (0.276)	−0.07 (395)
	OEA ^3^			1	0.52 (<0.001)	0.32 (<0.001)	0.02 (0.783)	0.02 (0.839)	0.02 (0.765)	−0.07 (0.369)	−0.13 (0.103)	−0.15 (0.064)	−0.00 (0.977)	0.05 (0.526)	−0.04 (0.614)
	UOE ^4^				1	0.58 (<0.001)	0.12 (0.118)	0.07 (0.411)	0.16 (0.042)	−0.27 (0.001)	−0.22 (0.006)	−0.38 (<0.001)	−0.22 (0.005)	−0.02 (0.819)	−0.07 (0.373)
	ROE ^5^					1	0.21 (0.007)	0.12 (0.122)	0.26 (0.001)	−0.33 (<0.001)	−0.27 (0.001)	−0.38 (<0.001)	−0.25 (0.001)	−0.20 (0.009)	−0.28 (<0.001)
CEP ^6^	Total						1	0.91 (<0.001)	0.91 (<0.001)	−0.46 (<0.001)	−0.61 (<0.001)	−0.30 (<0.001)	−0.24 (0.002)	−0.20 (0.009)	−0.28 (<0.001)
	CF ^7^							1	0.67 (<0.002)	−0.32 (<0.001)	−0.44 (<0.001)	−0.18 (0.024)	−0.17 (0.033)	−0.15 (0.052)	−0.23 (0.004)
	CQ ^8^								1	−0.52 (<0.001)	−0.67 (<0.001)	−0.37 (<0.001)	−0.28 (<0.001)	−0.22 0(.005)	−0.30 (<0.001)
AA ^9^	Total									1	0.62 (<0.001)	0.77 (<0.001)	0.79 (<0.001)	0.74 (<0.001)	0.37 (<0.001)
	FOP ^10^										1	0.45 (<0.001)	0.24 (0.003)	0.15 (0.052)	0.38 (<0.001)
	PC ^11^											1	0.53 (<0.001)	0.36 (<0.001)	0.12 (0.118)
	PA ^12^												1	0.57 (<0.001)	0.21 (0.008)
	FL ^13^													1	0.33 (<0.001)
Ageism															1

^1^ Emotional Intelligence. ^2^ Self-emotional appraisal. ^3^ Others’ emotional appraisal. ^4^ Use of emotion. ^5^ Regulation of emotion. ^6^ Contact with elderly people. ^7^ Contact frequency. ^8^ Contact quality. ^9^ Anxiety about aging. ^10^ Fear of old people. ^11^ Psychological concerns. ^12^ Physical appearance. ^13^ Fear of loss.

**Table 5:** Influencing factors on ageism among general hospital nurses. (*N* = 162).

Variables	Ageism					
	B	β	t	*p*	Tolerance	VIF
(constant)	1.55		9.58	<0.001		
AA-FOP ^1^	0.21	0.34	4.81	<0.001	0.98	1.02
AA-FL ^2^	0.15	0.28	3.89	<0.001	0.98	1.02
	R^2^ = 0.22, Adjusted R^2^ = 0.21, F = 22.56, *p* < 0.001					

^1^ Anxiety about aging—fear of old people. ^2^ Anxiety about aging—fear of loss. VIF: variance inflation factor.

## Data Availability

The data presented in this study are available on request from the corresponding author. The data are not publicly available due to participants’ privacy.
